# A cross sectional study on levels of dental anxiety, its influencing factors, and the preferred management techniques among patients in Riyadh, Saudi Arabia

**DOI:** 10.1371/journal.pone.0309248

**Published:** 2024-09-06

**Authors:** Albandri Mohammed Alowid, Mamata Hebbal, Alhanoof Aldegheishem, Varkey Nadakkavukaran Santhosh, Ram Surath Kumar, Atheer Mohammed Alfayyadh, Njoud Ibrahim Alateeq, Sara mazen Alomran, Shog Abdulelah Aleban

**Affiliations:** 1 Restorative Department, College of Dentistry, Princess Nourah Bint Abdulrahman University, Riyadh, Saudi Arabia; 2 Department of Preventive Dental Sciences, College of Dentistry, Princess Nourah Bint Abdulrahman University, Riyadh, Saudi Arabia; 3 Department of Clinical Dental Sciences, College of Dentistry, Princess Nourah Bint Abdulrahman University, Riyadh, Saudi Arabia; 4 Department of Public Health Dentistry, KLE V.K Institute of Dental Sciences, KLE Academy of Higher Education and Research, Belagavi, India; 5 College of Dentistry, Princess Nourah bint Abdulrahman University, Riyadh, Saudi Arabia; Danube Private University, AUSTRIA

## Abstract

**Background:**

Dental anxiety is marked by intense and irrational fear of dental procedures affecting millions of individuals worldwide. The purpose of this study was to investigate the relationship between dental anxiety, its influencing factors, and the preferred management techniques among adults seeking dental care in Riyadh.

**Methods:**

A cross-sectional questionnaire study was carried out among 1734 patients who visited dental clinics in Riyadh. A self-administered questionnaire was developed and validated, comprising 28 closed-ended questions; it demonstrated good reliability and internal consistency (Cohen’s kappa coefficient = 0.90, Cronbach’s alpha coefficient = 0.86), it contained pre-validated Modified Dental Anxiety Scale to quantitatively assess the level of dental anxiety. Data analysis involved descriptive analysis, Chi-square test, Pearson correlation coefficient and multiple linear regression.

**Results:**

Majority (59.2%) of participants reported moderate anxiety, while 10.9% experienced high anxiety which was significantly linked to factors such as fear of pain (37.8%) and anaesthetic needles (25.8%). Topical anaesthetic gel (64.5%), pre-treatment explanations (78.9%), and conducive clinic environment (79.4%) were perceived as effective anxiety alleviators. A negative correlation existed between dental anxiety and preferred management techniques. Dental anxiety had significant association between gender (β = 0.910) and age (β = 0.263).

**Conclusion:**

This study revealed that majority of participants had moderate dental anxiety, linked significantly to influencing factors like frequency and purpose of dental visits and past dental experiences. The study also found the preferred anxiety management methods among participants which included topical anaesthetic gel, pre-treatment explanations, and a comfortable clinic environment.

## Introduction

Dental anxiety, characterized by an extreme and irrational fear of dental procedures, is a concern impacting millions globally [1]. The global estimated pooled prevalence of dental fear and anxiety was notably high [2]. It transcends cultural and socioeconomic boundaries, posing a significant challenge in maintaining optimal oral health [3]. In the context of Saudi Arabia, the situation presents a comparable cause for concern. A previous study conducted in Jazan, Saudi Arabia, unveiled a high prevalence (80.8%) of dental anxiety among the participants [4]. A recent systematic review revealed that the incidence of dental anxiety in the central region of Kingdom of Saudi Arabia which included Riyadh was 31.6% [5]. This fear and anxiety not only leads to avoidance of dental visits and delay in seeking necessary treatment but also contributes to poorer oral health outcomes, perpetuating a cycle of anxiety and neglect [6].

Understanding the unique constellation of factors contributing to dental anxiety is crucial for addressing this issue effectively. Several specific factors are known to influence dental anxiety. Gender, age and past negative dental experiences, particularly involving pain or discomfort, can foster a lasting fear [7]. Additionally, dental anxiety may be heightened by elements such as apprehension in the waiting area, irregular patterns of dental attendance, invasive therapeutic interventions, compromised emotional well-being, deferment of dental visits due to anxiety, and residency in rural environments. Moreover, individual personality traits, like fearfulness or low pain tolerance, may play a role [8]. Hence, addressing the factors influencing dental anxiety is crucial because it that individuals receive necessary dental care without fear or hesitation.

Research consistently demonstrates that dental anxiety is a significant contributor to poor oral health outcomes. Individuals with high levels of dental anxiety exhibit higher prevalence of dental caries, untreated dental pathologies, and compromised periodontal status compared to non-anxious patients. This anxiety-driven avoidance of dental care often results in a cascade of negative oral health consequences [9]. This relationship is intensified by a "vicious cycle dynamic," in which apprehension of dental procedures, reduced utilization of dental services, and the prevalence of oral health ailments mutually reinforce one another [10]. Recognizing this intricate interplay, it becomes imperative to systematically address dental anxiety through targeted interventions and tailored support, aiming to alleviate psychological barriers and ultimately enhance oral health outcomes. The imperative to diminish dental anxiety is underscored by its pivotal role in enhancing patient compliance, satisfaction, and the likelihood of seeking dental care. Cognitive techniques, such as cognitive restructuring, have demonstrated notable efficacy in significantly reducing anxiety levels among phobic dental patients [11].

This study addresses a crucial knowledge gap by systematically examining the prevalence and determinants of dental anxiety among adults in Riyadh. It explores the frequency and reasons for dental visits, past experiences, and anticipated dental procedures, as well as therapeutic approaches for managing anxiety. By focusing on this geographical area, the study provides region-specific insights that can inform tailored interventions and strategies for reducing anxiety. These findings contribute valuable insights to the scientific literature by enhancing the understanding of dental anxiety within the Riyadh population. Thus, the objectives of this study were to determine the prevalence of dental anxiety, identify its key influencing factors, and explore the preferred management techniques for managing dental anxiety among patients seeking dental care in Riyadh.

## Materials and methods

### Study design and study setting

A cross-sectional observational study was carried out among 1734 dental patients who visited both public and private dental clinics. Three public dental centres were randomly selected from 65 public dental centres and four private dental clinics were randomly selected from 284 dental private dental centres in the Riyadh province of Kingdom of Saudi Arabia. (Data based on ministry of health latest statistics of total numbers of dental centre in Riyadh region, General Directorate Of Heath affairs In Riyadh). The data collection was done between October 2022 to January 2023.The present study adhered to the STrengthening the Reporting of OBservational studies in Epidemiology (STROBE) guidelines.

### Ethical consideration and informed consent

This study received approval from the Research Ethics Committee at Princess Nourah bint Abdulrahman University IRB Log No: 22–0407, dated 25 August 2022. The study objectives were elucidated to all participants, and their explicit written informed consent was obtained. Participants who voluntarily provided consent were enrolled in the study.

### Sample size and sampling technique

Based on a previous study by Fayad et al. [12], the minimum sample size was estimated to be 1585 participants with a power of 0.95 and an alpha error of 0.05. A two-stage random sampling approach was employed to recruit the participants for inclusion in the study. Initially, from a pool of 236 dental clinics situated across 15 municipal districts in Riyadh province, five districts were randomly chosen [13]. Subsequently, in the second stage, five dental clinics were selected from each of the previously chosen districts. The final sample size of 1734 participants was established for this study.

### Instrument development and pilot testing

The study instrument underwent a comprehensive development process encompassing multiple stages. The initial stage involved the establishment of a conceptual framework, derived from an in-depth analysis of existing literature and insights from experts in the field. Subsequently, the questionnaire was meticulously structured into four key constructs. The first construct documented demographic variables such as gender, age, educational qualification, and income status of the participants. Following the demographic documentation, a pre-validated questionnaire, specifically the Modified Dental Anxiety Scale (MDAS) [14], was employed in the second construct to quantitatively assess the levels of dental anxiety among participants. The third construct was designed to elicit responses related to the various factors influencing dental anxiety. Finally, the fourth construct was dedicated to ascertaining the preferences of participants concerning anxiety management techniques.

In the second stage an item pool was generated for each construct. A focus group discussion was conducted involving both participants and the investigator, fostering a comprehensive exploration of the contents of the instrument. Cognitive interviewing was employed to delve into the understanding of participants regarding the items in the instrument. Throughout these stages, a continuous evaluation approach was implemented, leading to the judicious addition or removal of specific items based on their perceived relevance.

**Fig 1:**
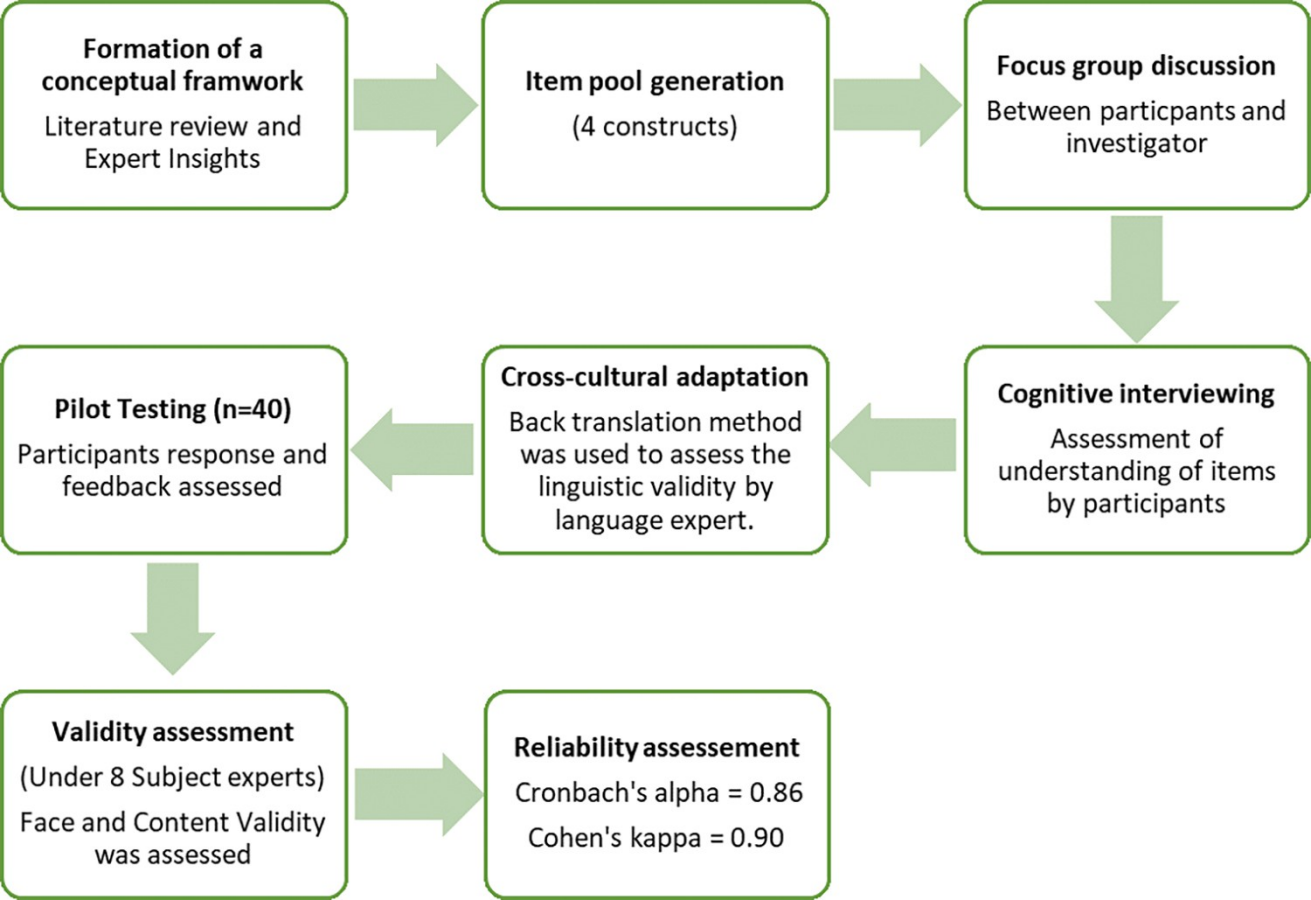
Steps involved in instrument development.

The study instrument was administered in both English and the regional language (Arabic), to ensure inclusivity. To assess linguistic validity, a back-translation process was employed, wherein the English version underwent translation into Arabic and subsequent validation by a language expert. An expert committee then undertook cross-cultural adaptation of the translated questionnaire, focusing on establishing semantic, idiomatic, experiential, and conceptual equivalence across the source and target versions within four distinct domains [15]. Pilot testing was carried out involving 40 participants to assess the appropriateness of the participants’ response and to determine the feasibility of the study. Following the pilot study, constructive feedback from participants was incorporated to refine and improve the questionnaire. Subsequently, face and content validity evaluation of the instrument took place with an expert committee consisting of eight subject matter experts. To gauge the instrument’s reliability, inter-rater and test-retest reliability were assessed using Kappa statistics. The computed Cohen’s kappa coefficient demonstrated a robust value of 0.90, signifying a substantial level of agreement. Additionally, an evaluation of internal consistency through Cronbach’s alpha coefficient yielded a value of 0.86, indicating good internal reliability within the instrument [Fig 1].

### Data collection procedure, and response grading

Data collection was carried out within the chosen dental clinics, three public and four private centers. The questionnaire was distributed to the participants [Google form link sheet] and the anticipated time required for each participant to complete the questionnaire was approximately 10 to 15 minutes. Participants with a higher educational background received the questionnaire in English, while those with a different educational profile were provided with the Arabic version. This approach was implemented to ensure the suitability of the instrument for all participants. The initial segment featured six multiple-choice questions. Subsequently, the MDAS scale, comprising five questions, utilized a response framework ranging from "not anxious" to "extremely anxious," each corresponding to scores from 1 to 5. The overall scale exhibited a scoring range from 5 to 25, with scores falling within the ranges of 5–9, 10–18, and ≥19 categorized as indicative of less, moderate, and high levels of dental anxiety, respectively [16]. The third segment of the questionnaire, dedicated to evaluating influencing factors, comprised six questions with responses recorded in the form of multiple-choice options. The final section of the questionnaire, focusing on preferred management techniques, included seven questions with responses categorized as "Effective”, “I don’t know” and "Not effective,", which were scored 2, 1 and 0, respectively. [S1 File]

### Data processing, analysis, and language enhancement

The collected data were entered into Microsoft Excel (2019) and subjected to analysis using IBM SPSS^®^ Statistics, Version 21.0 (IBM Corp., Armonk, NY, 2012). Descriptive statistics were presented, with categorical variables expressed as frequencies and percentages, while continuous variables were represented as mean ± standard deviation. To identify potential differences in demographic variables, Analysis of Variance (ANOVA) and independent t-test were employed. The Chi-square test was utilized to assess significant association among the study variables. Pearson correlation coefficient was calculated to determine associations between variables. Multiple linear regression analysis was conducted to explore relationship among various study variables. The level of statistical significance was set at p ≤ 0.05. AI-generated chatbot ChatGPT (version GPT-3.5) was used to improve the language and comprehension of the manuscript.

## Results

Among the 1734 participants, majority (79.6%) were females and belonged to the age group of 19–29 (35.6%) and 30–40 (30.1%). The majority possessed educational backgrounds at the undergraduate level (68.6%) and were almost evenly distributed among the income stream of < 5000 SR (30.3%), 5,000–10,000 SR (30.2%) and >10,000 SR (39.4%). Table 1 summarizes the demographic characteristics of the study participants.

**Table 1:** Comparison of the level of dental anxiety according to the gender, age, educational level, and income variables in the study (*N* = 1734).

Variable		*n* (%)	Dental Anxiety	Statistics
			Mean ± SD	*p*-value
Gender^α^	Male	353 (20.4)	11.03 **±** 4.19	< .001*
	Female	1381 (79.6)	12.92 **±** 4.56	
Age^¥^	19–29 years	618 (35.6)	12.02 **±** 4.56^a^	0.006*
	30–40 years	522 (30.1)	12.77 **±** 4.38^bc^	
	41–60 years	519 (29.9)	12.87 **±** 4.66^cd^	
	>60 years	75 (4.3)	12.77 **±** 4.55^abcd^	
Educational level^¥^	Primary school** **	18 (1.0)	11.83 **±** 5.78^a^	0.308
	Secondary school, Postsecondary education	353 (20.4)	12.87 **±** 4.68^a^	
	Higher education (Undergraduate)	1190 (68.6)	12.49 **±** 4.47^a^	
	Postgraduate	173 (10.0)	12.16 **±** 4.65^a^	
Income^¥^	< 5000 SR	526 (30.3)	12.68 ± 4.68^a^	0.293
	5,000–10,000 SR	524 (30.2)	12.66 ± 4.45^a^	
	>10,000 SR	684 (39.4)	12.32 ± 4.52^a^	
	Total	1734 (100)	12.53 **±** 4.55	

1SR (Saudi Riyal) = 0.35 US$ [Purchasing Power Parity (PPP) adjusted to United States Dollar (Currency reference year 2024)]. All values are expressed as mean ± standard deviation (SD) and frequency with percentages (in parentheses). The statistical test used

^¥^ One-way ANOVA and ^α^ Independent t-test; Level of significance

*p ≤ 0.05 is considered statistically significant.

### Dental anxiety level

**Fig 2:**
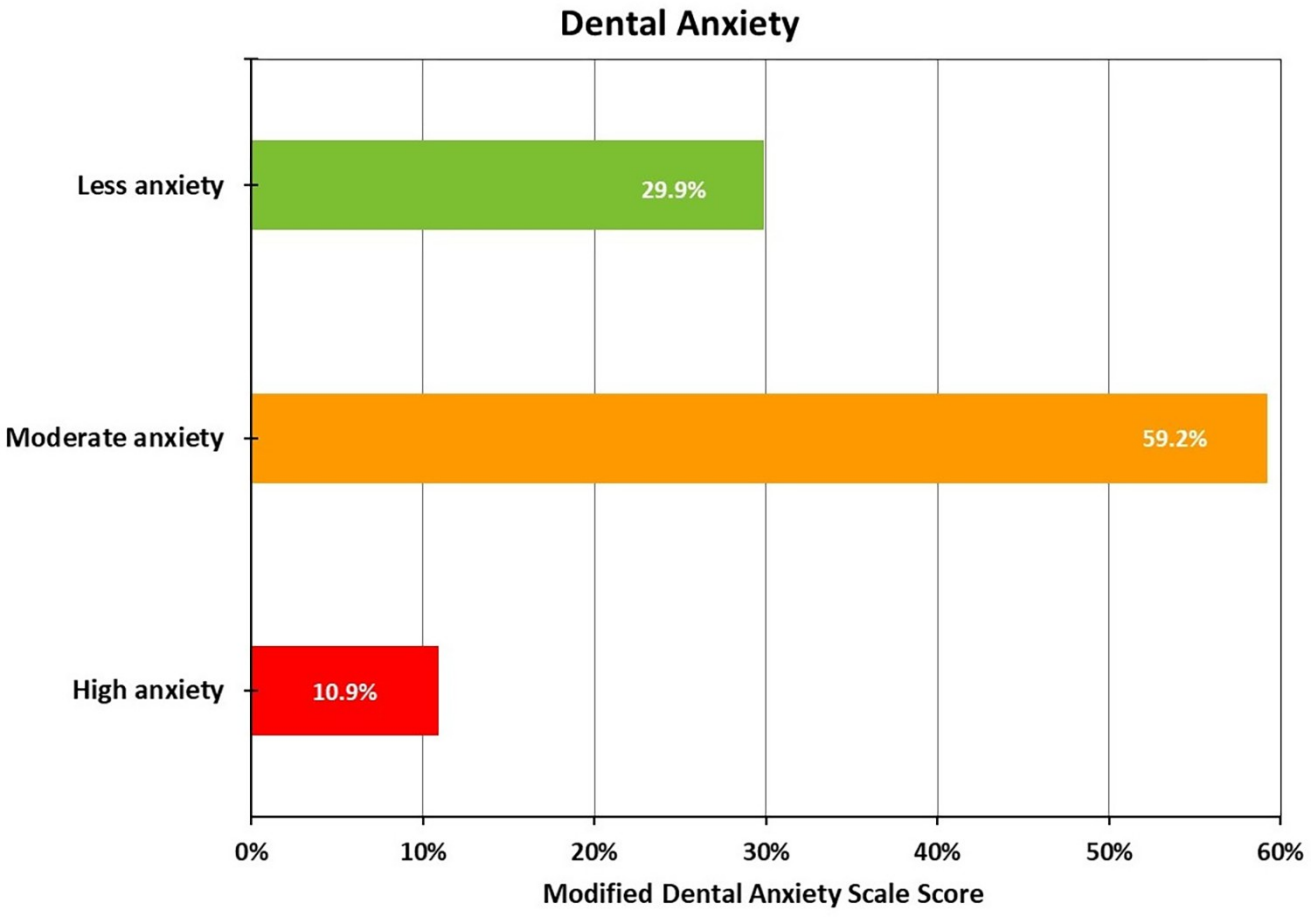
Distribution of patients according to their dental anxiety level.

MDAS scale revealed that the mean dental anxiety score for the participants was 12.53 ± 4.55. Majority of the participants (59.2%) had moderate level of dental anxiety with the score of 13.50 ± 2.45, while high level of anxiety was observed in 10.9% of the participants [Table 2 and Fig 2].

**Table 2:** Distribution of patients according to their dental anxiety level (*N* = 1734).

Modified Dental Anxiety Scale Score	*n* (%)	Mean ± SD
Less anxiety	519 (29.9)	7.49 ± 1.36
Moderate anxiety	1026 (59.2)	13.50 ± 2.45
High anxiety	189 (10.9)	21.13 ± 1.89
Total	1734 (100.0)	12.53 ± 4.55

All values are expressed as mean ± standard deviation (SD) and frequency with percentages (in parentheses).

### Influencing factors of dental anxiety

There was a statistically significant association observed between dental anxiety and the various responses to the questions toward the duration and frequency of dental visits, the reason for their visit, past dental experiences, and the nature of the dental procedure planned. The participants primarily visited the dentist for pain (29.1%) followed by cosmetic treatments (18.1%); p < .001. The major reason for anxiety during dental treatment was due to fear of pain (37.8%) followed by anaesthetic needle (25.8%); p < .001. Participants predominantly felt the physical symptom of palpitations (30%); p < .001 and felt most fear while receiving the dental treatment (46.7%); p < .001. Among the dental procedures, root canal treatment induced the highest level of anxiety among participants (39.9%), closely followed by extraction (32.2%); p < .001 [Table 3].

**Table 3:** Association between the total dental anxiety score and responses to questions regarding its influencing factors.

Question		Response Frequencies	Statistics
		*n* (%)	*p*-value
How many times have you visited the dentist in the last two years?	Once in the last two years	303 (17.5)	0.379
	Twice in the last two years	514 (29.6)	
	More than three times	917 (52.9)	
How long did your last visit to dentist take?	Less than 30 minutes	608 (35.1)	0.001*
	30–60 minutes	803 (46.3)	
	More than 60 minutes	323 (18.6)	
What type of sector did you visit?	Governmental dental sector	336 (19.4)	0.448
	Private dental sector	1048 (60.4)	
	Both	350 (20.2)	
What is the qualification of your dentist which you have majorly visited?	Undergraduate students	107 (6.2)	0.003*
	Postgraduate students	79 (4.6)	
	General practitioner	169 (9.7)	
	Specialist	505 (29.1)	
	Consultant	487 (28.1)	
	I don’t know	387 (22.3)	
What is the reason for your current visit to the dentist?	Dental pain (nerve treatment, sensitivity, abscess)	504 (29.1)	< .001*
	Extraction	163 (9.4)	
	Cosmetic treatment (fillings and crowns)	313 (18.1)	
	Implants	118 (6.8)	
	Orthodontic treatment	167 (9.6)	
	Cleaning	245 (14.1)	
	Regular checkup	80 (4.6)	
	Other reasons	144 (8.3)	
What is the reason for fear of receiving dental treatment?	Anesthesia needle	448 (25.8)	< .001*
	Fear of pain	655 (37.8)	
	Presence of blood/doctor’s tools	67 (3.9)	
	Sound of the drilling tool	294 (17.0)	
	Not applicable	270 (15.6)	
How did the fear of the dentist begin?	Unknown reason	676 (39.0)	< .001*
	Previous bad experience	490 (28.3)	
	Lack of Doctor empathy	56 (3.2)	
	From others experiences	100 (5.8)	
	Not applicable	412 (23.8)	
What physical symptoms do you feel during dental treatment?	Tremors	330 (19.0)	< .001*
	Headedness	111 (6.4)	
	Sweating	147 (8.5)	
	Palpitations	521 (30.0)	
	Not applicable	625 (36.0)	
When do you feel your fear is at peak?	One day before the date of the visit	155 (8.9)	< .001*
	At the waiting room	286 (16.5)	
	While receiving treatment	810 (46.7)	
	After treatment	21 (1.2)	
	Not applicable	462 (26.6)	
Which of the following treatments causes the most anxiety?	Cleaning	64 (3.7)	< .001*
	Fillings	83 (4.8)	
	Extraction	559 (32.2)	
	Implants	153 (8.8)	
	Root canal treatment	691 (39.9)	
	Other	58 (3.3)	
	Not applicable	126 (7.3)	

All values are expressed as the frequency with percentages (in parentheses). The total dental anxiety score was used as ordinal data. The statistical test used: Chi-square test; Level of significance

*p ≤ 0.05 is considered statistically significant.

### Preferred management techniques in reducing dental anxiety

The participants overwhelmingly perceived that the application of topical anaesthetic gel before needle injection (64.5%), providing an explanation of the treatment prior to commencing any dental procedure (78.9%), and creating a conducive environment in the dental clinic (79.4%), were effective in alleviating dental anxiety (p < .001). A significant proportion (44.9%) expressed the opinion that music was not effective in reducing dental anxiety (p < .001). A substantial number of participants (42.6%) were uncertain about the effectiveness of 3D glasses in reducing dental anxiety; p < .001, [Table 4]. A statistically significant association was observed between responses regarding preferred management techniques to reduce dental anxiety and responses related to its influencing factors (p < .05).

**Table 4:** Responses to the questions toward preferred management techniques in reducing dental anxiety.

Question	Response Frequencies *n* (%)			Statistics
	Effective	Not effective	I don’t know	*p*-value
Do you agree that the topical anaesthetic gel before needle injection is effective in reducing dental fear?	1119 (64.5)	142 (8.2)	473 (27.3)	< .001*
Do you agree that conscious sedation /general anaesthesia is effective in reducing dental fear?	749 (43.2)	361 (20.8)	624 (36.0)	0.225
Do you agree that relaxation techniques such as music are effective in reducing dental fear?	511 (29.5)	779 (44.9)	444 (25.6)	< .001*
Do you agree that distraction methods such as 3D glasses are effective in reducing dental fear?	675 (38.9)	321 (18.5)	738 (42.6)	< .001*
Do you agree that explain the treatment from the dentist before starting the procedure are effective in reducing dental fear?	1368 (78.9)	176 (10.1)	190 (11.0)	< .001*
Do you agree that the clinic environment has a role in reducing dental fear?	1377 (79.4)	100 (5.8)	257 (14.8)	< .001*
Do you agree that anti-anxiety medications are effective in reducing dental fear?	552 (31.8)	286 (16.5)	896 (51.7)	0.068

All values are expressed as the frequency with percentages (in parentheses). The statistical test used: Chi-square test; Level of significance

*p ≤ 0.05 is considered statistically significant.

### Relationship between demographic variables and dental anxiety/preferred management techniques

There was negative correlation which was statistically significant seen between dental anxiety (MDAS score) and preferred management techniques in reducing dental anxiety (p < 0.001) [Table 5].

**Table 5:** Correlation between dental anxiety and the responses to the questions toward therapeutic ways to deal with anxiety.

Variable	Preferred management techniques in reducing dental anxiety	
Dental anxiety (MDAS)	*r*	-0.15
	*p*	< .001*

The statistical test used: Pearson correlation coefficient test; level of significance

*p ≤ 0.05 is considered a statistically significant correlation

Abbreviation: MIDAS, Modified Dental Anxiety Scale

Multiple linear regression analysis was performed with dental anxiety score and preferred management techniques in reducing dental anxiety as dependent variables and predictors such as gender, age, educational level and income status of the participants [Table 6]. There was statistically significant relationship observed between dental anxiety and predictors such as gender (β = 0.910; p < .001) and age (β = 0.263; p = .001). Similar relationship was observed between preferred management techniques and predictors [gender (β = -0.285; p < .001) and age (β = -0.306; p < .001)].

**Table 6:** Results of multiple linear regression on factors associated with dental anxiety and the responses to the questions toward preferred management techniques in reducing dental anxiety.

Dependent variable	Independent variable	β	SE	*p*-value	95% CI for β
Dental anxiety (MDAS)	Constant	10.475	0.406	< .001*	9.680–11.271
	Gender (Male)	0.910	0.136	< .001*	0.643–1.177
	Age (ref: 19–29 years)	0.263	0.082	0.001*	0.102–0.425
	Educational Level (ref: Postgraduate)	0.033	0.119	0.780	-0.200–0.266
	Income (ref: >10,000 SR)	0.092	0.133	0.487	-0.168–0.352
Preferred management techniques in reducing dental anxiety	Constant	10.522	0.239	< .001*	10.054–10.990
	Gender (ref: Male)	-0.285	0.080	< .001*	-0.442-(-0.128)
	Age (ref: 19–29 years)	-0.306	0.048	< .001*	-0.401-(-0.211)
	Educational Level (ref: Postgraduate)	-0.041	0.070	0.556	-0.178–0.096
	Income (ref: >10,000 SR)	-0.044	0.078	0.575	-0.197–0.109

β: regression coefficient; SE: standard error; CI: confidence interval; the statistical test used: Multiple linear regression analysis model; level of significance

*p ≤ 0.05 is considered statistically significant relationship.

## Discussion

Dental anxiety significantly influences oral health by avoidance behaviours, leading to delayed or deferred dental appointments. This avoidance pattern often precipitates the exacerbation of dental conditions and undermines proactive preventive measures, contributing to a cascading effect on the overall oral health [17]. In addition, the heightened psychological distress experienced during dental procedures may impede effective patient-dentist communication, potentially diminishing the precision and thoroughness of oral health interventions [18]. Hence, this cross-sectional study in Riyadh, Saudi Arabia, was aimed at unravelling the relationship between dental anxiety, its influencing factors, and the preferred management techniques for managing dental anxiety among adults seeking dental care.

In this study, comprising 1734 participants, a notable majority (79.6%) were females. Previous studies by Jeddy *et al*.[19], and Dadalti *et al*.[20], reported a higher degree of dental anxiety among females. This indicates that gender is an important factor that influences dental anxiety. Majority of the participants in this study were young and middle-aged adults who had at least undergraduate level of education belonging to various streams. The impact of age on dental anxiety is evident, with studies suggesting that younger individuals often experience higher levels of anxiety compared to their older counterparts, possibly due to unfamiliarity with dental procedures [21]. Including middle-aged adults is crucial as they may experience unique stressors affecting dental anxiety and have different management preferences, providing a comprehensive view of dental anxiety across adulthood [12]. The level of education plays a pivotal role, as individuals with higher educational attainment tend to exhibit improved comprehension and coping mechanisms, potentially mitigating dental anxiety [22]. Furthermore, economic disparities can impact access to dental care, with those in lower-income brackets encountering obstacles that may intensify anxiety when attending dental appointments [22]. Gender, age, education, and socio-economic status significantly impact dental anxiety, with younger individuals, lower education levels, and lower socio-economic status often experiencing higher levels of apprehension due to limited awareness, potential financial constraints, and varied exposure to oral health education [21]. Higher education and socio-economic status are generally associated with better dental health literacy and resources, contributing to lower levels of anxiety among those demographics [22]. The rigorous methodology used to develop the study instrument ensures the validity and reliability. By integrating a comprehensive conceptual framework and employing methods like cognitive interviewing, pilot testing, and linguistic validation, the questionnaire effectively captures dimensions of dental anxiety while ensuring cultural sensitivity [23]. High inter-rater reliability and internal consistency values further affirm the instrument’s precision.

Participants in this study predominantly exhibited a moderate level of dental anxiety. These findings diverge from those reported by AlDhelai *et al*., [4] and Sivaramakrishnan *et al*., [24] in Saudi Arabia, where lower levels of dental anxiety were observed. Global studies revealed a lower occurrence of dental anxiety in various countries. For instance, in France, only 6.2% of adults encounter moderate dental anxiety [25], whereas the United States reports a prevalence of 19% for moderate anxiety [26]. Similarly, Norway demonstrates a lower prevalence of high dental anxiety at 4.2% [27], while Denmark reports 6% of adults with moderate dental anxiety [22]. These disparities may be attributed to cultural, socioeconomic, and healthcare-related factors influencing the perception and experience of dental anxiety across diverse populations. The moderate level of dental anxiety observed in patients visiting dental clinics in Riyadh can be attributed to a range of factors. These include fear of pain, previous negative dental experiences, and anxiety related to anaesthetic injections and surgical procedures [12]. Additionally, socioeconomic factors like limited access to dental services, concerns about treatment costs, and time constraints contribute to irregular dental visits [12]. Unique to the region of Saudi Arabia, the cultural practice of Khat chewing which also contributes to a role in shaping dental anxiety [28]. Addressing these multifaceted factors through education, improved accessibility, and culturally sensitive care can help alleviate anxiety and enhance oral health outcomes in Riyadh.

The present study investigates the relationship between dental anxiety and influencing factors, revealing the intricate interplay of psychological and environmental variables in shaping dental anxiety. A significant association was observed between dental anxiety and the duration of the last dental visit. Jamali *et al*., reported a consistent trend of deteriorating behaviour among children as the treatment duration increased, highlighting the impact of time on the overall dental experience [29]. Hence, shorter appointments can significantly reduce dental anxiety. Majority of participants reported experiencing fear during dental treatment, aligning with findings from Dou *et al*., where majority encountered fear during endodontic procedures [30]. The primary reason participants visited the dentist was pain, accompanied by a predominant fear stemming from the anticipation of pain and the origin of pain was unknown to them. These results were consistent with previous studies conducted by Wijk *et al*. [31] and Halling *et al*.[32], highlighting a parallel pattern in the association between dental visits, fear, and pain perception. Majority of participants reported experiencing palpitations during dental treatment, with heightened anxiety particularly noted during extraction and root canal procedures. These observations were consistent with findings by Russel [33] and Pieterse *et al* [34].

The present study also documented the preferred management techniques for dental anxiety among the participants. In this study, the majority of participants reported that the application of topical anaesthetic gel before injection was effective in reducing dental fear. Study by Agarwal *et al*. [35], indicated the efficacy of topical anaesthetic gel in alleviating pain during needle insertion for local anaesthesia. Participants predominantly expressed that receiving explanations from the dentist about the treatment before initiating the procedure was effective in mitigating dental fear. This aligns with the findings from a study by Soh., demonstrating that furnishing relevant details of treatment procedures significantly reduced dental fear in apprehensive patients [36]. Additionally, a study by Ng *et al*., reported that pre-operative information regarding both operative procedures and expected recovery led to a substantial reduction in self-reported anxiety among patients undergoing surgical dental procedures [37]. Similarly, the environment of the dental clinic had a significant role as majority felt it helps in reducing dental fear, this aligned with the findings reported by Shapiro *et al*.[38], however, it contradicted those of Fux-Noy et al.,[39]. The participants in this study were unsure of the effectiveness of 3D glasses and anti-anxiety medications in reducing the fear. However previous studies indicated that these methods aids in reducing the dental fear [40, 41]. Majority of participants perceived music as an ineffective solution in reducing dental fear. A randomized controlled trial conducted by Wazzan *et al*. also reported that music did not contribute to a reduction in dental anxiety [42]. Although these techniques are found to be effective, equally crucial are dentist behaviours characterized by empathy, friendliness, and projecting a calm and competent image, all of which play pivotal roles in anxiety reduction and the augmentation of patient satisfaction. An enhanced understanding of the multifaceted causes of dental anxiety, coupled with the implementation of effective management techniques, further contributes to the improvement of anxiety levels [43].

This study yields valuable insights into the intricate landscape of dental anxiety among residents of Riyadh. The identification of a prevalence of moderate anxiety levels, examination of specific influencing factors, and determination of preferred management techniques emphasize the imperative for personalized and patient-centered approaches in dental care. Uniquely, this research combines three key parameters: dental anxiety prevalence, preferred management techniques, and influencing factors among dental patients in Riyadh, Saudi Arabia. Additionally, the study’s large sample size enhances the reliability and generalizability of the findings. Dental practitioners in Riyadh should consider demographic variables, individual fears, and preferences when formulating anxiety management strategies.

The cross-sectional design of this study restricts its ability to examine the long-term impact of these factors on dental anxiety. It prevents the establishment of a temporal relationship between various influencing factors and dental anxiety. Future research investigations could examine additional influencing factors, such as cultural aspects and previous traumatic experiences, to further enhance our understanding of dental anxiety in Riyadh. Longitudinal studies, monitoring changes in anxiety levels and responses to interventions over time, could provide deeper insights into the dynamics of dental anxiety. The implementation of targeted interventions, including customized communication strategies and innovative anxiety reduction techniques, based on demographic variables and individual preferences, has the potential to significantly enhance the dental care experience for patients in Riyadh. By fostering a comprehensive understanding of dental anxiety, healthcare providers can cultivate a more supportive and patient-friendly environment, ultimately increases the dental care utilization in the community and promotes better oral health outcome.

## Conclusion

The study revealed a significant prevalence of moderate dental anxiety among patients in Riyadh. It established the associations between dental anxiety and influencing factors such as the duration and frequency of dental visits, the purpose of the visit, prior dental experiences, and the anticipated nature of the dental procedure. Additionally, preferred management techniques for dental anxiety were found to be the use of topical anaesthetic gel, pre-treatment explanations, and creating a conducive clinic environment. These findings emphasize the need for personalized and patient-centered approaches in dental care to effectively manage dental anxiety.

## Supporting information

S1 File The questionnaire used in the study. (PDF)
