# Supplementary material for: A cross sectional study on levels of dental anxiety, its influencing factors, and the preferred management techniques among patients in Riyadh, Saudi Arabia
Source: PLoS One. 2024 Sep 6;19(9):e0309248. doi: 10.1371/journal.pone.0309248 (PMC11379177; doi:10.1371/journal.pone.0309248)
Supplement: S1 File — (PDF) [file pone.0309248.s001.pdf]

IRB Log Number: 22-0407

This study aims to explore the extent of anxiety related to dental treatment, to identify the factors influencing anxiety associated with dental treatment, and the preferred management techniques among adults seeking dental care in Riyadh, Saudi Arabia.

Kindly filling out the questionnaire (it will not take you more than 5 minutes)

Section A: (General Questions)

Q1. Nationality:

1. Saudi
2. Non-Saudi

Q2. Place of residence:

1. Riyadh
2. Other

Q3. Gender:

1. Male
2. Female

Q4. Age:

1. 19-29
2. 30-40
3. 41-60
4. 60<

Q5. Educational level:

1. Primary school
2. Secondary school, Postsecondary (tertiary) education
3. Higher education (bachelor's degree)
4. Postgraduates

Q6. Income:

A- Less than 5000 SR
B- 5,000-10,000 SR
C- More than 10,000 SR

Q7. How many times have you visited the dentist in the last two years?

1. 1

2. 2-3
3. More than 3

Q8. How long did your last visit to dentist take?

1. Less than 30 minutes
2. 30-60 minutes
3. More than 60 minutes

Q9. What type of sector did you visit?

1. Governmental dental sector
2. Private dental sector
3. Both

Q10. What is the qualification of your dentist?

1. Undergraduate students
2. Postgraduate students
3. General practitioner
4. Specialist
5. Consultant
6. I don't know

Section B: (Modified Dental Anxiety Scale)

*Can you tell us how anxious you are, if any, from your visit to the dentist? *

Q11. If you went to the dentist tomorrow, how would you feel?

A- Calm, not anxious
B- Slightly anxious
C -Fairly anxious
D- Very anxious
E- Extremely anxious

Q12. If you were sitting in the waiting room (waiting for treatment), how would you feel?

A- Calm, not anxious
B- Slightly anxious
C -Fairly anxious
D- Very anxious
E- Extremely anxious

Q13. If the dentist started drilling on your teeth, how would you feel?

A- Calm, not anxious
B- Slightly anxious
C -Fairly anxious

D- Very anxious
E- Extremely anxious

Q14. If the dentist started cleaning and polishing your teeth, how would you feel?

A- Calm, not anxious
B- Slightly anxious
C -Fairly anxious
D- Very anxious
E- Extremely anxious

Q15. If you were about to get a local anesthetic injection, how would you feel?

A- Calm, not anxious
B- Slightly anxious
C -Fairly anxious
D- Very anxious
E- Extremely anxious

Section C: (Fear and Anxiety Questions)

Q16. What is the reason for your current visit to the dentist?

1. Dental pain (nerve treatment, tooth sensitivity, abscess)
2. Extraction
3. Cosmetic treatment (filling, crowns)
4. Implants
5. Orthodontic treatment
6. Cleaning
7. Regular checkup
8. Other reasons

Q17. What is the reason for fear of receiving dental treatment?

1. Anesthesia needle
2. Fear of pain
3. Presence of blood/doctor's tools
4. Sound of the drilling tool
5. Not applicable

Q18. How did the fear of the dentist begin?
1. Unknown Reason
2. Previous bad experience
3. Lack of doctor empathy
4. From other experiences
5. Not applicable

Q19. What physical symptoms do you feel during dental treatment?

1. Tremors
2. Headedness
3. Sweating
4. Palpitations
5. Not applicable

Q20. When do you feel your fear is at peak?

1. One day before the date of the visit
2. At the waiting room
3. While receiving treatment
4. After treatment
5. Not applicable

Q21. Which of the following treatment causes the most anxiety?

1. Cleaning
2. Fillings
3. Extraction
4. Implants
5. Root Canal treatment
6. Other
7. Not applicable

Section D: (Preferred Management)

Q22. Do you agree that the topical anesthetic gel before needle injection is effective in reducing dental fear?

1. Effective
2. Not effective
3. I don't know

Q23. Do you agree that conscious sedation /general anesthesia is effective in reducing dental fear?

1. Effective
2. Not effective
3. I don't know

Q24. Do you agree that relaxation techniques such as music are effective in reducing dental fear?

1. Effective
2. Not effective
3. I don't know

Q25. Do you agree that distraction methods such as 3D glasses are effective in reducing dental fear?

1. Effective
2. Not effective
3. I don’t know

Q26. Do you agree that explain the treatment from the dentist before starting the procedure are effective in reducing dental fear?

1. Effective
2. Not effective
3. I don’t know

Q27. Do you agree that the clinic environment has a role in reducing dental fear?

1. Effective
2. Not effective
3. I don’t know

Q28. Do you agree that anti-anxiety medications are effective in reducing dental fear?

1. Effective
2. Not effective
3. I don’t know

THANK YOU.
